# Supplementary material for: A UMLS-based spell checker for natural language processing in vaccine safety
Source: BMC Med Inform Decis Mak. 2007 Feb 12;7:3. doi: 10.1186/1472-6947-7-3 (PMC1805499; doi:10.1186/1472-6947-7-3)
Supplement: Additional file 13 — RAP application directory. Sets up directory for RAP (RDF application for PHP) source code [file 1472-6947-7-3-S13.gz › rap/doc/tests/Test_Storing_Models_in_Database.html]

Test Set Database Connection


### RDF API for PHP V0.9.1

# Test: Storing Models in Database

This example is part of the RAP - Rdf API for PHP documentation.

  

### 1. Connect to rdf database (e.g. Access via ODBC)

Set the system DSN for the particular database file (control pannel -> administrative
tools -> data sources (odbc) -> system DSN).  
If you want to use other databases read: Set
Database Connection.

`// Include RAP  
define("RDFAPI_INCLUDE_DIR", "./../api/");  
include(RDFAPI_INCLUDE_DIR . "RDFAPI.php");  
   
// Connect to MsAccess database (rdf_db DSN)   
// using connection settings defined in constants.php :  
$rdf_database = ModelFactory::getDbStore();`

  
  

### 2. Store a memory model in rdf database

Load an RDF-Documtent into a memory model

`// Filename of an RDF document  
$base="example1.rdf";  
  
// Create a new memory model   
$memModel = ModelFactory::getDefaultModel();  
  
// Load and parse document  
$memModel->load($base);`

Now store the model in database

`// An unique modelURI will be generated  
$rdf_database->putModel($memModel);  
  
// You can also provide an URI for the model to be stored  
$modelURI = "example1.rdf";  
  
// But then you must check if there already is a model with the same modelURI  
// otherwise the method putModel() will return FALSE  
if ($rdf_database->modelExists($modelURI))   
   echo "Model with the same URI: '$modelURI' already exists";
  
else  
   $rdf_database->putModel($memModel, $modelURI);`

  
  

### 3. Create a new database Model

`$modelURI = "newDbModel";  
  
// Base URI of the new model (optional)  
$baseURI = "baseURIofMyNewDbModel#";  
  
// Get a new DbModel  
if ($rdf_database->modelExists($modelURI))   
   echo "Model with the same URI: '$modelURI' already exists";
  
else  
   $dbModel = $rdf_database->getNewModel($modelURI, $baseURI);`

  
  

### 4. List all models stored in rdf database

`// Get an array with modelURI and baseURI of all models stored
in rdf database  
$list = $rdf_database->listModels();  
  
// Show the database contents  
foreach ($list as $model) {  
   echo "modelURI: " .$model['modelURI'] ."<BR>";  
   echo "baseURI : " .$model['baseURI'] ."<BR><BR>";  
}`

**Output:**  
  
modelURI: DbModel-1  
baseURI : http://www.w3.org/Home/Lassila.rdf#  
  
modelURI: example1.rdf  
baseURI : http://www.w3.org/Home/Lassila.rdf#  
  
modelURI: newDbModel  
baseURI : baseURIofMyNewDbModel#
